# Limited independent prognostic value of MMP-14 and MMP-2 expression in ovarian cancer

**DOI:** 10.1186/s13000-016-0485-3

**Published:** 2016-04-02

**Authors:** M. Caroline Vos, Anneke A. M. van der Wurff, Johan Bulten, Roy Kruitwagen, Harrie Feijen, Toin H. van Kuppevelt, Thijs Hendriks, Leon F. A. G. Massuger

**Affiliations:** Department of Obstetrics and Gynaecology, Elisabeth Tweesteden Hospital, PO Box 90151, 5000 LC Tilburg, The Netherlands; Department of Pathology, Elisabeth Tweesteden Hospital, PO Box 90151, 5000 LC Tilburg, The Netherlands; Department of Pathology, Radboud University Nijmegen Medical Centre, PO Box 9101, 6500 HB Nijmegen, The Netherlands; Department of Obstetrics and Gynaecology, Tweesteden Ziekenhuis, PO Box 90107, 5000 LA Tilburg, The Netherlands; Present address: Department of Obstetrics and Gynaecology and GROW – School for Oncology and Developmental Biology, Maastricht University Medical Centre, Maastricht, The Netherlands; Department of Obstetrics and Gynaecology, Amphia Hospital, Langedijk 75, 4819 EV Breda, The Netherlands; Department of Biochemistry, Radboud Insitute for Molecular Life Sciences, Radboud University Medical Center, PO Box 9101, 6500 HB Nijmegen, The Netherlands; Department of Surgery, Radboud University Nijmegen Medical Centre, PO Box 9101, 6500 HB Nijmegen, The Netherlands; Department of Obstetrics and Gynaecology, Radboud University Nijmegen Medical Centre, PO Box 9101, 6500 HB Nijmegen, The Netherlands

**Keywords:** MMP-2, MMP-14, Immunohistochemistry, Ovarian cancer, Prognosis

## Abstract

**Background:**

In cancer, various MMPs play a role in progression and metastasis and their overexpression generally indicates a poor prognosis. MMP-14 is the main activator of MMP-2 and both molecules play a role in normal ovarian follicular development. Earlier reports indicated a prognostic value for both MMP-14 and MMP-2 in ovarian cancer. This study was designed to determine the prognostic value of MMP-14 and MMP-2 expression in ovarian cancer with data on long-term follow-up.

**Methods:**

Tumor samples of 94 consecutive ovarian cancer patients from one regional laboratory were evaluated. Clinical and survival data were collected and related to known prognostic factors, as well as to the expression of MMP-14 and MMP-2 as determined by semi-quantitative immunohistochemistry.

**Results:**

Epithelial MMP-14 expression correlated with stromal MMP-14 expression (rho = .47, *p* < .01) and epithelial MMP-2 expression was found to correlate with both MMP-14 epithelial and stromal expression (rho = −.28, *p* < .01 respectively rho = −.21, *p* < .05). In univariable analysis of 64 advanced-staged tumours, no MMP parameter was significant for progression-free or overall survival. In multivariable analysis for PFS, stromal MMP-14 expression and epithelial MMP-2 expression remained in the model. For overall survival, no MMP parameter showed significance.

**Conclusions:**

We confirmed the correlation between epithelial and stromal MMP-14 expression and between epithelial MMP-2 and both epithelial and stromal MMP-14 expression. In this study with long-term follow-up, the independent prognostic value of MMP-14 and MMP-2 expression in ovarian cancer is limited to a role in PFS for stromal MMP-14 expression and epithelial MMP-2 expression.

## Background

An important distinguishing feature of ovarian cancer is the low survival rate of patients [1]. Screening is not possible, and because symptoms occur late in the disease process, most patients are diagnosed with advanced-stage disease. Although the majority of these patients respond well to first-line treatment, eventually only 43.5 % of them survive more than 5 years [1]. Several well-researched factors influence survival, including age, stage, histological subtype and residual tumor after debulking surgery [2].

In search for prognostic markers that could be useful in managing ovarian cancer, we focused on MMP-14 and MMP-2. Matrix metalloproteinases (MMPs) are zinc-dependent proteases that are involved in degrading the extracellular matrix in normal physiological processes, such as reproduction, embryonic development and tissue remodeling, as well as in disease processes, including arthritis and cancer [3]. In normal ovarian physiology, MMP-14, the first described membranebound MMP, is involved in follicular growth. Concurrently, it is also the main activator of MMP-2 or gelatinase A, which is found in large quantities in follicular fluid and the corpus luteum [4].

In cancer, MMP-14 is also the main activator of MMP-2, which is then secreted into the extracellular matrix [5]. In many types of cancer, various MMPs play a role in progression and metastasis [3]. In general, they are overexpressed in primary and metastatic tumors with a poor prognosis. Therefore, MMP inhibition seems to be an attractive approach in treating cancer. Classical MMP inhibitors, including Marimastat, have been studied with mixed results, and antibodies against MMP-14 are currently being investigated [6–8].

In ovarian cancer, overexpression of MMPs such as MMP-2, MMP-7, MMP-9, MMP-11 and MMP-14 has been reported and correlated with invasion and metastasis [6]. Using immunohistochemistry, high expression of MMP-14 has been demonstrated in ovarian clear-cell carcinomas. This histotype is known for it’s poor prognosis [9]. MMP-14 and MMP-2 also appear to be overexpressed in serous and mucinous malignant ovarian tumor epithelium, while benign and borderline tumors show lower levels of expression [10]. Overexpression of MMP-14 and MMP-2 is significantly associated with a shorter disease-specific survival in ovarian cancer [11], although Brun et al. found that this association lost its significance after Bonferroni correction [12]. Also, Trudel et al. found little independent prognostic value for MMP-14 expression [13]. Most of these studies were performed at large referral centres, which may have influenced the results due to selection bias, where the younger and fitter patients are referred more easily than the elderly and frail patients.

The aim of the present study is to evaluate the prognostic value of MMP-14 and MMP-2 expression in ovarian cancer, as determined by semi-quantitative immunohistochemistry, in a large regional cohort. In our cohort, long-term survival 10–15 years after initial diagnosis could be determined.

## Methods

### Patients

This retrospective cohort study included all 116 patients diagnosed with ovarian cancer at St. Elisabeth Hospital and Tweesteden Hospital, both in Tilburg, the Netherlands, and at Amphia Hospital, in Breda, the Netherlands, between January 1997 and December 2003. They were followed until March 1, 2013. Patients who had undergone neoadjuvant chemotherapy with secondary cytoreductive surgery were excluded (*n* = 11), because previous chemotherapy may have influenced MMP expression in the immunohistochemical results at the time of surgery [14]. Four patients were lost to follow-up because they emigrated. In seven patients, no residual tumor material was available, leaving 94 patients for analysis of MMP-14 and MMP-2 expression.

### Prognostic factors

Clinical prognostic factors were collected from the patients’ medical records. FIGO stage (I to IV), histology, and differentiation grade were categorized according to the World Health Organization (WHO) criteria, grade being assigned according to the observer’s impression of architectural and cytological features [15, 16]. All histopathological results and previous slides were reviewed by two pathologists (AAW, JB). If they disagreed, consensus was found between them. Serum levels of CA-125 were determined preoperatively. Ascites volume was determined at the time of collection or surgery. Debulking surgery was found to be optimal if the maximum diameters (length, width, or depth) of the individual residual tumour deposits were all less than 1 cm.

### Treatment

In accordance with uniform treatment guidelines, all patients underwent a staging laparotomy in the event of clinical early-stage ovarian cancer, or a debulking procedure in the event of advanced-stage ovarian cancer. In patients with FIGO stage Ia or Ib ovarian cancer with differentiation grade I, no adjuvant therapy was given. All other patients received 6 to 9 courses of adjuvant platinum-based chemotherapy.

### Outcome

The interval between diagnosis and detection of disease progression (progression-free survival, PFS) and between diagnosis and death (overall survival, OS) were considered as end points. PFS was defined as the time (months) until progression after partial response/stable disease, or the time until recurrence after complete response. Disease progression was established if clinical symptoms (re-)appeared and/or ultrasound or computed tomography showed that recurrent tumor lesions had appeared or increased, often accompanied by a rise in CA 125. Patients who showed progression during first-line treatment were coded as having a PFS of 0 months. Information on the date of death and cause of death was derived from medical charts, hospital-registered death certificates, and death registrations by the patient’s general practitioner. Patients who had neither progressed nor died before March 1, 2013 were censored at the last follow-up date. If patients had died, but not from ovarian cancer, they were censored at the time of death.

### Semiquantitative immunohistochemistry

From the archives of the histopathology laboratory, paraffin-embedded blocks were selected. Semiquantative immunohistochemistry was performed as described before [17]. In short, after deparaffinisation in xylene and hydration in graded alcohol, sections of 3 μm were used. Each slide included positive controls (placenta). For blocking of endogenous peroxidase, 3 % H_2_O_2_ and 5 % normal goat serum was used. Slides were washed three times with phosphate-buffered saline (PBS) after each incubation step. As the primary antibody for MMP-2, a monoclonal antibody was used (clone A-Gel vc2, Thermo Scientific) [18] Incubation was in a dilution 1: 10 overnight at 4 °C. As the primary antibody for MMP-14, a polyclonal antibody (Thermo Scientific) [5] was used in a dilution 1:20 for 60 min at room temperature. As the secondary antibody, poly-HRP-GAM/R/R IgG Powervision (Immunologic, Duiven, the Netherlands) was used for 60 min at room temperature. Each run contained negative controls (i.e. without primary antibody). Staining was done twice with diaminobenzidine (Immunologic, Duiven, the Netherlands) in substrate buffer (20 μl) for 5 min. Counterstaining was performed with hematoxylin.

The scoring system that was used incorporated both the intensity of the scoring (0 = absent, 1 = weak, 2 = moderate, 3 = strong) and the percentage of positive tumor cells (0 = 0 %, 1 = 1–25 %, 2 = 26–50 %, 3 = 51–75 %, 4 = 76–100 %). Stromal staining was recorded separately as no, weak or strong staining. Points for the intensity and the percentage of staining were added and assigned an Overall Score according to Kamat [11]. The Overall Score was dichotomized in 0 for no-to-weak expression (1–2 points) and 1 for moderate to-strong expression (3–6 points). Two investigators (MCV, AAW) scored all slides for MMP staining. If they disagreed, consensus was reached between them.

### Statistical analysis

Statistical analysis was performed using the Statistical Package for Social Sciences 20.0 (SPSS Inc., Chicago, Il). Patient characteristics and correlations, as well as median PFS and OS, were computed and using the Kaplan-Meier method, survival curves were plotted. As early-stage patients have a relatively favourable prognosis, Cox regression survival analyses were performed for traditional clinicopathological and MMP variables only in the advanced-stage group of 64 patients. To correct for the relatively small sample size the Cox regression analysis was bootstrapped 1000 times in order to detect potential significance for MMP variables. A model was built using a backward selection procedure in Cox regression analysis, where all variables were manually deleted step by step for p values greater than 0.10. In this model, the traditional clinical prognostic factors – i.e. age, FIGO stage, histology, differentiation and cytoreduction – were included together with MMP-14 and MMP-2 Overall Scores and stromal staining. The use of more variables than these would violate the number-of-events-per-variable rule.

## Results

The patient characteristics are summarized in Table 1. Median follow-up time was 59 months (range 0–195 months). The cohort consisted of all consecutive patients from a regional laboratory, including 30 early-stage patients. All the patients were treated according to the guidelines that were in effect at the time of treatment (1997-2003).

**Table 1:** Patient characteristics

	Early stage (*n* = 30)		Advanced stage (*n* = 64)	
	Median (range)	Frequency	Median (range)	Frequency
Age	50 (25–73)		59 (31–88)	
Histology				
*Serous*		5		32
*Mucinous*		13		2
*Endometroid*		4		14
*Clear cell*		3		4
*Adenocarcinoma unspecified*		3		12
*Mixed*		2		0
Differentiation				
*Low-grade*		16		8
*High-grade*		14		56
CA 125	24 (0–4325)		321 (0–44438)	
Ascites				
*Absent*		23		15
*Present*		7		49
FIGO stage				
*IIB*				11
*III*				44
*IV*				9
Cytoreduction (only advanced stage disease)				
*Optimal*				35
*Suboptimal*				29
Progression Free Survival	116 (0–195)		17 (0–191)	
Overall Survival	120 (0–195)		34 (0–191)	

**Fig. 1:**
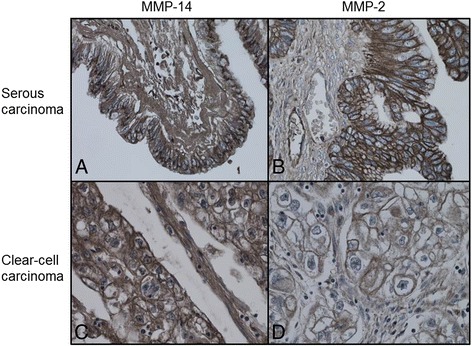
MMP-14 and MMP-2 staining. **a** MMP-14 staining of serous adenocarcinoma. **b** MMP-2 staining of serous adenocarcinoma. **c** MMP-14 staining of clear-cell carcinoma. **d** MMP-2 staining of clear-cell carcinoma

MMP-14 and MMP-2 expression was found in most of the tumours investigated, not only in the epithelium, but in a proportion of tumours also in the stroma. Results of MMP-staining are shown in Table 2. Staining was mainly pericellular for MMP-2 and mainly both cytoplasmatic and pericellular for MMP-14 if staining was epithelial (Fig. 1). Stromal staining was mainly diffuse. This is illustrated in Fig. 1, which shows representative slides of both MMP-14 and MMP-2 staining. Clearly, the stroma of a serous carcinoma stains positive for MMP-14 (1a) and negative for MMP-2 (1b). A case of clear-cell carcinoma shows intense expression for MMP-14 (1c) and less intense expression for MMP-2 (1d).

**Table 2:** MMP-14 expression and MMP-2 expression (numbers of patients)

	Early stage (*n* = 30)	Advanced stage (*n* = 64)
MMP-14 Overall Score (number of patients)		
*0*	10	31
*1*	20	33
MMP-14 stroma (number of patients)		
*No*	12	26
*Weak*	3	4
*Strong*	15	34
MMP-2 Overall Score (number of patients)		
*0*	2	9
*1*	28	55
MMP-2 stroma (number of patients)		
*No*	18	47
*Weak*	2	4
*Strong*	10	13

*MMP* Matrix Metalloproteinase

Seven patients in the whole cohort had a clear-cell carcinoma, which is known for it’s high level of MMP-14 expression: of these patients only one patient showed no epithelial and stromal MMP-14 staining and no MMP-2 stromal staining; epithelial MMP-2 staining however, was present in this patient. Six other patients with a clear-cell carcinoma showed epithelial MMP-14 staining of which two had no stromal MMP-14 staining. For MMP-2, the same pattern was observed in these two patients i.e. epithelial MMP-2 staining without stromal MMP-2 staining. Only one patient with a clear-cell carcinoma had both epithelial and stromal MMP-14 and MMP-2 expression. Nor epithelial MMP-14 and MMP-2 expression, nor stromal MMP-14 and MMP-2 expression was related to stage or survival in these seven patients with a clear-cell carcinoma. In all other histotypes, patients with both high and low expression of MMP-14 and/or MMP-2 in the epithelial and stromal compartment were found.

MMP-14 Overall Score correlated with MMP-14 in stroma and with MMP-2 Overall Score. The correlation coefficient of .47 between MMP-14 Overall Score in the tumour epithelium and MMP-14 in stroma indicates a strong relation. Also, MMP-14 in stroma and MMP-2 Overall Score correlated. The Spearman correlation coefficients between MMP-14 Overall Score and MMP-2 Overall Score and between MMP-2 Overall Score and MMP-14 in stroma, which were .28 respectively .21, indicate a moderate effect (Table 3).

**Table 3:** Spearman correlations between MMP-14 and MMP-2 expression

	MMP-14 OS	MMP-14 stroma	MMP-2 OS
MMP-14 Overall Score			
MMP-14 stroma	.47**		
MMP-2 Overall Score	.28**	.21*	
MMP-2 stroma	0.03	0.1	0.04

*MMP* Matrix MetalloProteinase

**. Correlation is significant at the 0.01 level (2-tailed)

*. Correlation is significant at the 0.05 level (2-tailed)

**Fig. 2:**
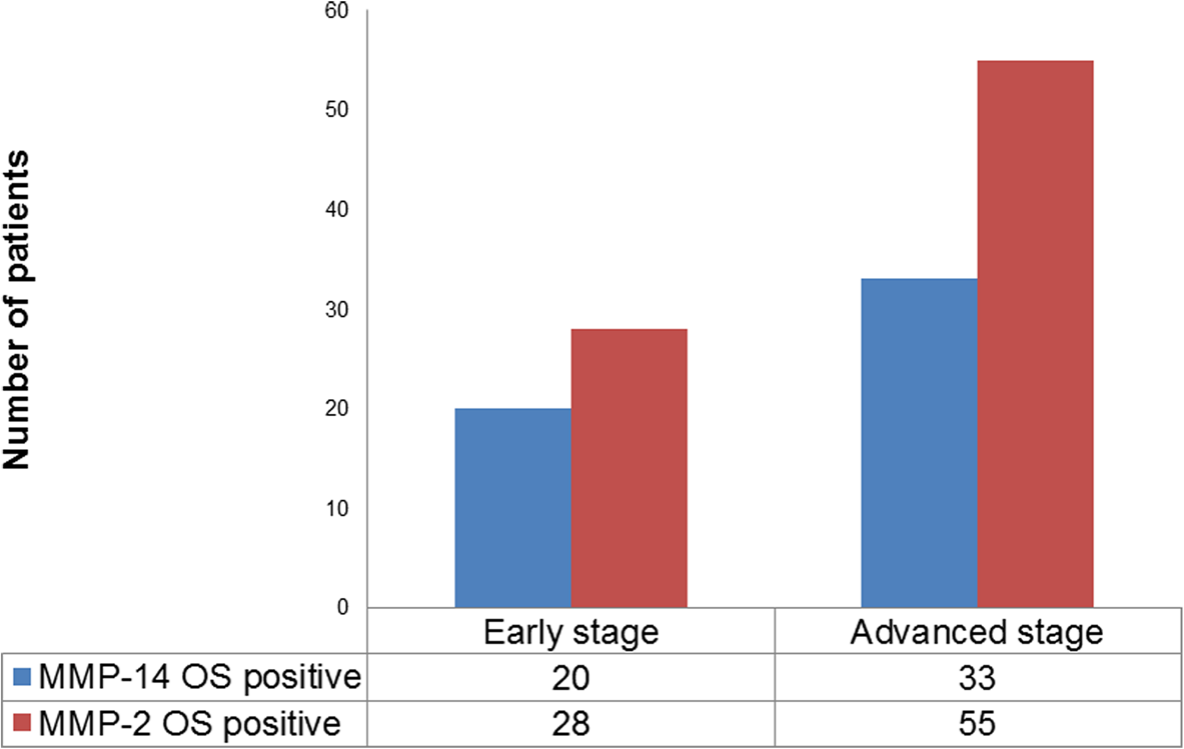
MMP-14 and MMP-2 Overall Score by FIGO stage. On the Y-axis the number of patients is given. OS = Overall Score

Considering the FIGO stage of patients, positive Overall Score for MMP-14 and MMP-2 differed between the two proteins. MMP-2 expression frequency is high in both early- and advanced-stage patients (28/30 respectively 55/64), while MMP-14 expression is less often present in both groups (20/30 respectively 33/64). See Fig. 2.

As Table 4 shows, univariable Cox regression analysis for PFS in 64 advanced-stage patients demonstrated a trend for MMP-14 stromal expression (HR 1.421 (95 % Confidence Interval 0.99–2.039, *p* 0.057). For overall survival, only age, histology and FIGO stage were significant after such analysis. None of the other MMP-14 or MMP-2 related parameters showed correlation with either PFS or OS in univariable analysis. Since bootstrapping our univariable Cox regression analysis did not result in new significant factors, it was decided to present the results without bootstrapping. In a multivariable Cox regression model in advanced-stage patients, aimed at identifying prognostic factors, age (*p* < .05), differentiation (*p* < .07), FIGO stage (*p* < .02), cytoreduction (*p* < .10), MMP-14 in stroma (*p* < .01) and MMP-2 Overall Score (*p* < .03) remained in the model for PFS. After bootstrapping, only FIGO stage (*p* < .04) and MMP-14 in stroma (*p* < .06) remained in the model for PFS. In multivariable analysis for OS, age (*p* < .00), histology (*p* < .00) and FIGO stage (*p* < .00) remained significant. After bootstrapping, only age (*p* < .03) and FIGO stage (*p* < .03) remained in the model for OS. In our group of nine FIGO IV patients, three patients were long-term survivors in spite of their FIGO IV stage disease. This may explain the lack of significance for this group in multivariable analysis.

**Table 4:** Hazard ratio's, 95 % confidence intervals and significance for progression-free survival and overall survival of advanced stage patients (significant factors are shown in bold)

	Progression free survival				Overall survival			
	HR	95,0 % CI for HR		Sig.	HR	95,0 % CI for HR		Sig.
		Lower	Upper			Lower	Upper	
Age	1.019	0.986	1.053	0.266	1.028	1.003	1.054	**0.03**
Histology				0.641				**0.003**
*Mucinous versus serous*	1.134	0.498	2.583	0.765	1.223	0.605	2.471	0.575
*Endometroid versus serous*	0	0	.	0.983	21.448	3.539	130.006	0.001
*Clear-cell versus serous*	0.542	0.187	1.575	0.261	0.559	0.228	1.369	0.203
*Adenocarcinoma unspecified versus serous*	1.099	0.231	5.226	0.906	1.335	0.369	4.835	0.66
Differentiation	3.266	0.778	13.716	0.106	1.702	0.674	4.297	0.26
CA 125								
*<35*				0.362				0.635
*35–200*	0.57	0.216	1.499	0.254	0.847	0.391	1.831	0.672
*>200*	0.62	0.254	1.515	0.295	0.709	0.34	1.478	0.359
Ascites	1.919	0.836	4.401	0.124	1.75	0.877	3.489	0.112
FIGO								
*IIB*				0.076				**0.044**
*III*	0.311	0.077	1.26	0.102	0.294	0.101	0.854	0.024
*IV*	1.06	0.371	3.028	0.914	0.839	0.389	1.807	0.653
Cytoreduction	1.882	0.939	3.774	0.075	1.696	0.935	3.076	0.082
MMP-14 Overall Score	1.538	0.777	3.043	0.216	0.945	0.551	1.624	0.839
MMP-14 stroma	1.421	0.99	2.039	0.057	0.695	0.371	1.299	0.254
MMP-2 Overall Score	22.901	0	#######*	0.668	0.879	0.395	1.953	0.751
MMP-2 stroma	1.214	0.807	1.828	0.352	1.15	0.832	1.59	0.399

*HR* Hazard Ratio, *CI* Confidence Interval, *Sig.* Significance, *MMP* Matrix Metalloproteinase

* = because of the low number of patients 95 % CI can not be calculated

**Fig. 3:**
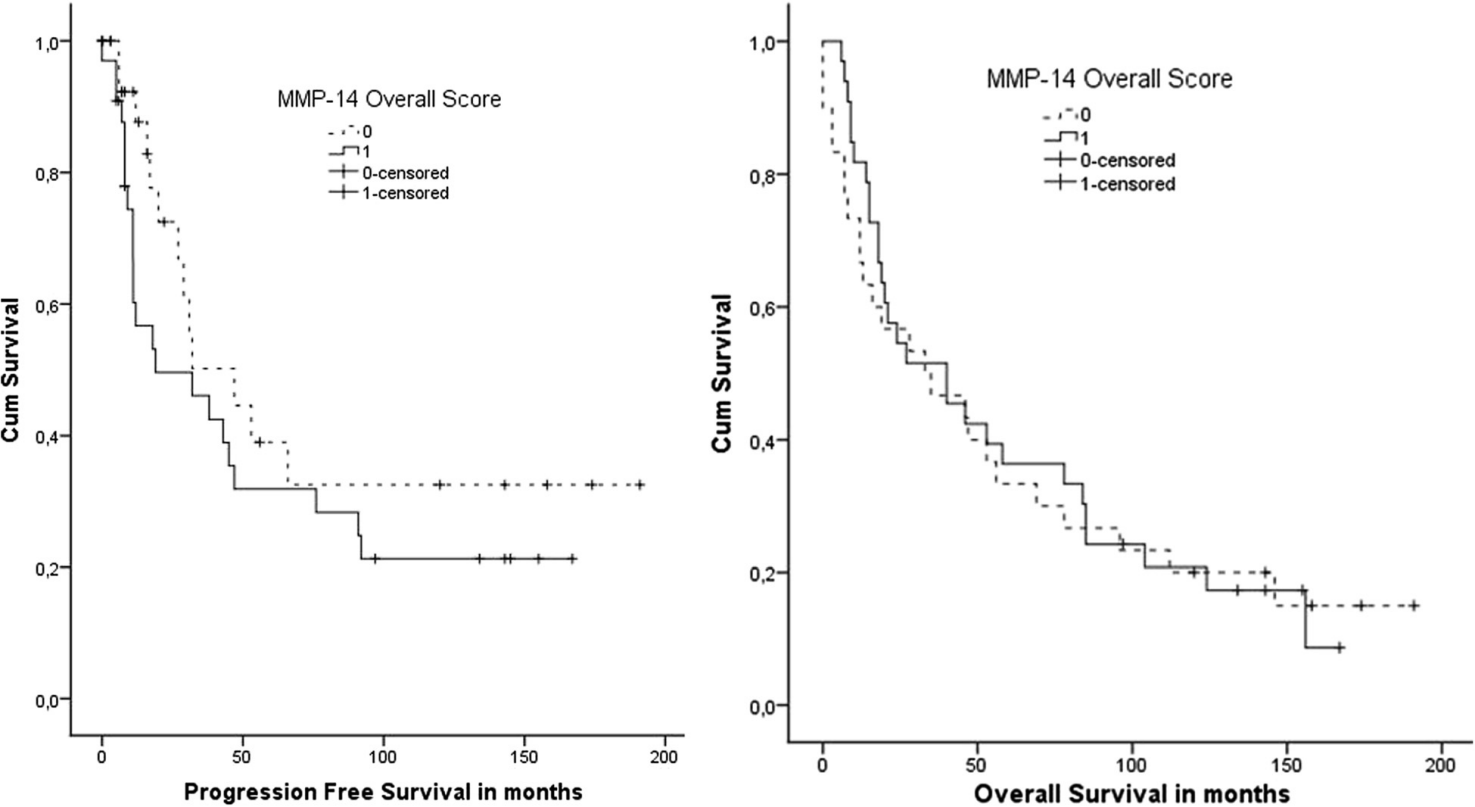
Kaplan-Meier curves of PFS and OS for advanced-stage patients by MMP-14 Overall Score

Figure 3 shows Kaplan-Meier curves for MMP-14 Overall Score for PFS and OS in advanced-stage patients, illustrating the lack of significance of MMP-14 expression in PFS or OS.

## Discussion

The findings in this study are in contrast with the previously reported promising prognostic values for MMP-14 and MMP-2 in ovarian cancer [11, 19], but underline the later reports by Brun et al. [12] and Trudel et al. [13] This large retrospective cohort study with long-term follow-up has shown a correlation between epithelial MMP-14 and MMP-2 expression, and for both with stromal MMP-14 expression. No MMP parameter has been found to be a significant prognosticator for progression-free survival or overall survival in univariable analysis, while known clinical and histopathological prognostic parameters (age, histology and FIGO stage) were for overall survival. In multivariable analysis for PFS, MMP-14 stromal staining and MMP-2 epithelial staining remained in the model. For overall survival, no MMP parameter was found to be a prognosticator.

The strengths of the present study are the use of a regional cohort with long-term follow-up and the use of immunohistochemistry in a diagnostic laboratory facility. Due to the use of a regional cohort, it is unlikely that the results of present study are strongly influenced by patient selection. The long-term follow-up eliminates bias by short-term outcomes. By using immunohistochemistry in a diagnostic laboratory facility, bias due the lack of reproducibility of our results is unlikely.

A possible limitation of the present study is the exclusion of the patients who were treated with neo-adjuvant chemotherapy, but this is only a small group in this cohort.

The magnitude of the correlations between the presence of MMP-14 in the tumour epithelium and MMP-14 in the tumour stroma indicate a strong effect. At the base of the invadopodia of the tumour cell, the activation of MMP-2 by MMP-14 takes place, so the observed correlation between MMP-14 and MMP-2 that has been found in other studies, is confirmed in our study [3, 5].

The comparison between the results of this and other studies on prognosis can be focussed on three factors: technical or procedural differences, tumor biology and/or selection of patients with different tumor biology for the studies.

With respect to techniques and procedures used in the different studies, the following observations can be made.

Similarly to the present study, all other studies that are cited here made use of immunohistochemistry. In addition, comparable primary antibodies were used, i.e. polyclonal antibodies for MMP-14 and a monoclonal antibody for MMP-2 [9–13]. Immunohistochemical results may also be influenced by differences in storage conditions of the tissue blocks [20]. All the cited studies used material collected over a lengthy time period (5–20 years). These technical aspects are comparable between the different studies and probably not the explanation for the differences between the results. However, differences in fixation protocols between the laboratories may explain the differences in results.

The second possible explanation for differences in results is tumor biology. The first factor to consider in this context is histologic subtype. As we did, Adley et al. found high MMP-14 expression in clear-cell carcinomas [6]. Expression of MMP-14 in serous and mucinous malignant epithelial cells has also been found [10]. In our cohort, MMP-14 and MMP-2 expression are not confined to clear-cell carcinomas, and not all clear-cell carcinomas express both proteins. Therefore, the differences in prognostic value of MMP-14 are not only attributable to histological subtype or selection by histological subtype.

Tumor stage is the following important factor in tumour biology and also an important prognosticator. In our cohort, high MMP-14 expression was found in two thirds of early-stage tumours whereas in the advanced-stage group only in half of the tumours high expression was found. For MMP-2, we found high expression not only in early-stage but also in advanced-stage tumors. A third possible explanation for the difference in results is patient selection, where patients with different prognosis may be overrepresented in some studies.

Kamat et al. investigated a series of 90 patients by means of immunohistochemistry and found a correlation between both epithelial and stromal MMP-14 expression and prognosis. As 90 % of their patients had advanced-stage disease, the expression pattern that they found may mainly reflect advanced-stage pathophysiology [11]. Also, they found stromal expression in 87 of their 90 patients, whereas we found such expression in only 52 of our 94 patients. Therefore, the difference between the Kamat series and our data may be due to selection of the patients, their series was collected at a large referral centre.

In line with our results, Brun et al. also found no independent prognostic value for MMP-14 and MMP-2 expression [12]. Their series comprised only advanced-stage tumours, with higher median age, CA 125 and percentage of interval debulking than in the present study. Trudel et al. investigated 211 tumor arrays after debulking surgery, where higher MMP-14 expression was associated with factors of better ovarian carcinoma prognosis. However, after correction for the known prognostic factors no prognostic value was found [13]. These results are more in accordance with our results than are the results from Davidson et al. [19] and Kamat et al. [11].

## Conclusion

Having investigated a regional cohort of 94 ovarian cancer patients by means of semiquantitative immunohistochemistry for MMP-14 and MMP-2, we found that epithelial MMP-14 and epithelial MMP-2 expression correlate. Furthermore, both correlate also with stromal MMP-14. In our cohort, no MMP parameter was significant in univariable analysis for progression-free survival in advanced-stage patients. In multivariable analysis for PFS, MMP-14 stromal expression and MMP-2 epithelial expression remained in the model with five other factors. For overall survival, no MMP parameter was significant in uni- or multivariable analysis. Thus the independent prognostic value of MMP-2 and MMP-14 expression in ovarian cancer is limited.

## Ethics approval and consent to participitate

The study was approved by the Medical Research Ethics Committee of the St. Elisabeth Hospital (P1009). The committee considered formal testing under the Medical Research Involving Human Subjects Act not necessary and the study was conducted under the Agreement on Medical Treatment Act. Therefore, formal informed consent of the individual patients was not necessary.

## Abbreviations

FIGO: Federation Internationale de Gynaecologie et Obstetrie
MMP: matrix metalloproteinase
OS: overall survival
PFS: progression free survival
PBS: phosphate-buffered saline
WHO: World Health Organisation
